# A Case of Extra-Uterine Pregnancy—Death—Autopsy

**Published:** 1884-02

**Authors:** Edward Clark

**Affiliations:** Post Mortem Examiner


### A Case of Extra-Uterine Pregnancy—Death—Autopsy.

By Edward Clark, M. D., Post Mortem Examiner.

A short time since, I was requested, by one of the county coroners, to make an autopsy on the remains of a woman who had died quite suddenly at her home on Elm street, in this city. On reaching the house, the attending physician was present, who gave me the following brief history of the case: He had been called to see her a short time before her death. She was suffering from intense abdominal pains, with all the symptoms of collapse. He administered morphia to quiet the pain. She continued to sink rapidly, and died in a short time. Not being able to form a positive opinion as to the cause of death, and the fact that a rumor that she had been hurt, prevailed, made him hesitate about issuing a death certificate. Thus the case fell into the coroner's hands, and I had the opportunity to make an autopsy. When the abdomen was opened, a large amount of fluid gushed out, that looked like liquor amnii, which undoubtedly it was, as will be presently seen. Continuing, I found a large blood clot that had spread itself out between the omentum and abdominal wall. I removed the clot and carefully turned up the omentum, and was not a little surprised to find a fœtus, apparently of about five months' intra-uterine development, lying face downward upon the coils of intestine. Hardly expecting to find a rupture of the uterus at so early a period of pregnancy, I continued my examination, curiously impatient to learn just what I should find. Raising up the fœtus and following down the umbilical cord, my hand was led into what seemed to be a large laceration or rent, apparently posterior to the uterine fundus, from which the placenta was partially protruding. I removed the fœtus, cord and placenta, and then proceeded to extirpate the uterus and its ligaments, together with a sac or cyst, which was attached to the uterus and broad ligament posteriorly, and to the right side. After its removal, I found the uterus enlarged and thickened, but perfectly intact. On section it was found to contain a well-developed decidua. In fact, the uterus and its contents indicated that nature had made every effort to have these parts keep pace with the development of the extra-uterine fœtus. The left ovary was in a natural condition; the right had either been obliterated by the fœtal sac in its growth, or else formed a part of its wall, I could not determine which. The sac from which the fœtus had escaped was firmly attached to the posterior part of the uterus and the broad ligament of the right side, and considerable force was necessary to sever it from its attachments to portions of the intestines. The most interesting question connected with extra-uterine pregnancy is that of diagnosis and treatment. If the nature of the case could be early recognized, it might be treated as an ordinary cystic growth of the ovary, or the fœtus could be destroyed by the electric current. But is it possible to make a positive diagnosis after rupture of the sac occurs? If so, would we have time to operate before death? Lastly, is an operation advisable, or is death certain to occur in such a case as the one here reported? I would like to hear from some of my professional confreres in regard to the questions here presented.

